# Optical Contrast and Raman Spectroscopy Techniques Applied to Few-Layer 2D Hexagonal Boron Nitride

**DOI:** 10.3390/nano9071047

**Published:** 2019-07-22

**Authors:** Marie Krečmarová, Daniel Andres-Penares, Ladislav Fekete, Petr Ashcheulov, Alejandro Molina-Sánchez, Rodolfo Canet-Albiach, Ivan Gregora, Vincent Mortet, Juan P. Martínez-Pastor, Juan F. Sánchez-Royo

**Affiliations:** 1 Instituto de Ciencia de Materiales, Universidad de Valencia (ICMUV), P.O. Box 22085, 46071 Valencia, Spain; 2 Institute of Physics, Academy of Sciences Czech Republic v.v.i, Na Slovance 1999/2, 18221 Praha 8, Czech Republic

**Keywords:** Hexagonal boron nitride, two-dimensional materials, optical contrast, Raman spectroscopy

## Abstract

The successful integration of few-layer thick hexagonal boron nitride (hBN) into devices based on two-dimensional materials requires fast and non-destructive techniques to quantify their thickness. Optical contrast methods and Raman spectroscopy have been widely used to estimate the thickness of two-dimensional semiconductors and semi-metals. However, they have so far not been applied to two-dimensional insulators. In this work, we demonstrate the ability of optical contrast techniques to estimate the thickness of few-layer hBN on SiO_2_/Si substrates, which was also measured by atomic force microscopy. Optical contrast of hBN on SiO_2_/Si substrates exhibits a linear trend with the number of hBN monolayers in the few-layer thickness range. We also used bandpass filters (500–650 nm) to improve the effectiveness of the optical contrast methods for thickness estimations. We also investigated the thickness dependence of the high frequency in-plane E_2g_ phonon mode of atomically thin hBN on SiO_2_/Si substrates by micro-Raman spectroscopy, which exhibits a weak thickness-dependence attributable to the in-plane vibration character of this mode. Ab initio calculations of the Raman active phonon modes of atomically thin free-standing crystals support these results, even if the substrate can reduce the frequency shift of the E_2g_ phonon mode by reducing the hBN thickness. Therefore, the optical contrast method arises as the most suitable and fast technique to estimate the thickness of hBN nanosheets.

## 1. Introduction

Two-dimensional (2D) materials attract a lot of attention for their electronic and optoelectronic applications at the nanoscale due to their outstanding physical properties, differing from their bulk state. The electronic nature of 2D materials prepared so far ranges from metallic to insulating. The best known 2D materials include graphene, transition metal dichalcogenides (TMDs), black phosphorus (BP) and hexagonal boron nitride (hBN). hBN is an electrical insulator with a large band gap. It consists of boron and nitrogen atoms arranged like graphene in a hexagonal honey-comb lattice structure. Each boron atom is covalently bonded through sp^2^ hybridization to three neighboring nitrogen atoms with a bond length of 1.45 Å [1,2]. Individual layers of hBN lattice are coupled by weak van der Waals forces with an interlayer spacing of 0.333 nm [2]. The most preferable stacking order of atoms was found to be AÁ [3]. Stacking faults can introduce crystal point defects such as a boron atom composition in a pyramidal or sp**^3^** configuration instead of a planar one [3]. Other not yet well-known defects presenting in the crystal lattice are impurities (O, C, Si…) and vacancies [4,5]. Both stacking faults and impurities could affect its optoelectronic properties and could lead to creation of single photon emitters [6,7]. The surface of hBN is atomically smooth without dangling bonds and charge traps and it has optical phonon modes [1,2,5]. It has a band gap of 5.955 eV and an exciton binding energy of about 130 meV [8,9]. Regarding calculations, the high band gap of hBN is direct for a monolayer and it becomes indirect for a larger number of monolayers and bulk [10,11]. Experimentally, a residual p-type character of typical h-BN was found, because the maximum valence band is located near the K point, 2.5 eV below the Fermi level [12]. hBN also has a high Young’s modulus (≈ 270 N m^−1^) and thermal conductivity (up to 400 W m^−1^ K^−1^ at room temperature) [2]. The pristine hBN is transparent in the visible region, but it absorbs and emits light at the deep ultraviolet region [2,13,14].

Integration of ultra-thin hBN into 2D van der Waals heterostructures opens the door for fabrication of new nano-devices, such as field-effect transistors (FETs) [15,16,17,18] and thermoelectric devices [19] with an enhanced performance [5,20]. Due to its high chemical stability, hBN is also an ideal material for the passivation of air-unstable 2D materials, such as BP or some TMDs, to protect them against ambient degradation [20,21,22,23,24]. Defective hBN can be also used for an indirect doping of BP [25]. Recently, hBN has been proposed as a potential material for electrochemical sensing of dopamine [26,27]; when covered by metal nanoparticles, it can be used for surface enhanced Raman spectroscopy (SERS) [28]. Its excellent photoluminescence properties with a bright single photon emission at UV [13,14], visible and near infrared regions after a treatment [6,7], make hBN a promising candidate for quantum technologies. 

The most common technique for 2D hBN synthesis is mechanical exfoliation (cleavage) from its bulk crystal state by using scotch tape and polydimethylsiloxane (PDMS) as substrates [29]. Another technique is liquid phase exfoliation using polar solvents to break forces between individual layers of the crystal. These techniques achieve exfoliated crystals of a few micrometers lateral size with a random number of layers. A large sized hBN with controllable thickness up to atomic level can be synthetized by chemical vapour deposition (CVD) [30] or by magnetron sputtering [31]. Despite the advantages of these techniques, mechanical exfoliation is still the fastest and cheapest method to synthetize good quality 2D materials.

Thickness identification of 2D materials is essential for their characterization and the implementation of several applications requiring different nanosheet thicknesses at precise sites (accessible by transfer micro-manipulation). Many techniques for counting the number of layers exist, such as atomic force microscopy (AFM) [32] and transmission electron microscopy (TEM) [33]. The physical properties of few-layer 2D crystals are thickness dependent and the number of layers can be identified by photoluminescence (PL) measurements [34,35]. Raman spectroscopy is another useful technique to distinguish the number of layers of 2D materials [36,37]. Optical contrast (OC) determination is the fastest and cheapest non-destructive technique for thickness identification. It is based on the different intensity of reflected light from an oxidized substrate and 2D material standing on this substrate by using white [38,39,40,41] or monochromatic [42,43] illumination through an optical microscope and simple software to extract the optical contrast.

In this work, we demonstrate experimentally and theoretically that thickness identification of mechanically exfoliated hBN crystals is possible on standard SiO_2_/Si substrates (285 nm ± 10 nm of oxide thickness) by OC. Given that hBN exhibits very low OC in comparison to other 2D materials, we propose enhancing its magnitude by using illumination near-monochromatic light, efficiently achieved in this work by using color bandpass filters. We verified that the OC-technique is fast and accurate for the thickness determination of hBN nanosheets, because near-monochromatic OC exhibits a linear dependence with the thickness of nanosheets up to around 50 monolayers. We also compared the OC capabilities to determine the thickness of few-layer hBN with other optical techniques, such as micro-Raman spectroscopy. In this way, we investigated the high frequency in-plane E_2g_ phonon mode of atomically thin hBN crystals. The experimental results were supported by ab initio calculations of the Raman active phonon modes of atomically-thin free standing crystals. Experimentally and theoretically, the frequency of the E_2g_ phonon mode increases as the number of hBN monolayers reduces, but only below 3–4 monolayers. Furthermore, we found a difference in the maximum frequency shift of this Raman mode from bulk to near-2D between experimental (6 cm^−1^) and calculated (4 cm^−1^) values, which is attributed to the nanosheet corrugation induced by the substrate [44]. As a conclusion of this study, the OC-method is comparatively more powerful and simple than Raman for a fast determination of the thickness of hBN nanosheets.

## 2. Materials and Methods 

Few-layer hBN crystals were prepared by mechanical exfoliation of commercially available hBN single crystals from HQ Graphene by a scotch tape and transferred onto (285 ± 10 nm) SiO_2_/Si substrates. The substrates were cleaned by ultrasonic bath in acetone, isopropanol and water followed by ozone cleaning. An Eclipse LV150A optical microscope from Nikon equipped with a Nikon DSFI2 high-definition color camera was used for optical image acquisition. For visible light illumination we used four different bandpass filters centered at wavelengths 500, 550, 600, and 650 nm with a transparent window of ∼40 nm. Experimentally, the OC was extracted from optical images [38], and the theoretical values were calculated by solving Fresnel equations. In order to calibrate and validate our method applied to hBN crystals, the thickness was confirmed by atomic force microscopy (AFM) measurements carried out at room temperature on an ambient AFM (Bruker, Dimension Icon) in Peak Force Tapping mode with ScanAsyst Air tips (Bruker; *k* = 0.4 N/m; nominal tip radius 2 nm). Micro-Raman spectroscopy using a Renishaw InVia system was carried out at room temperature using a blue laser illumination with 488 nm excitation wavelength, 7.5 mW laser power and a 50× objective. 

## 3. Results and Discussions

### 3.1. Optical Contrast Calculations

**Figure 1:**
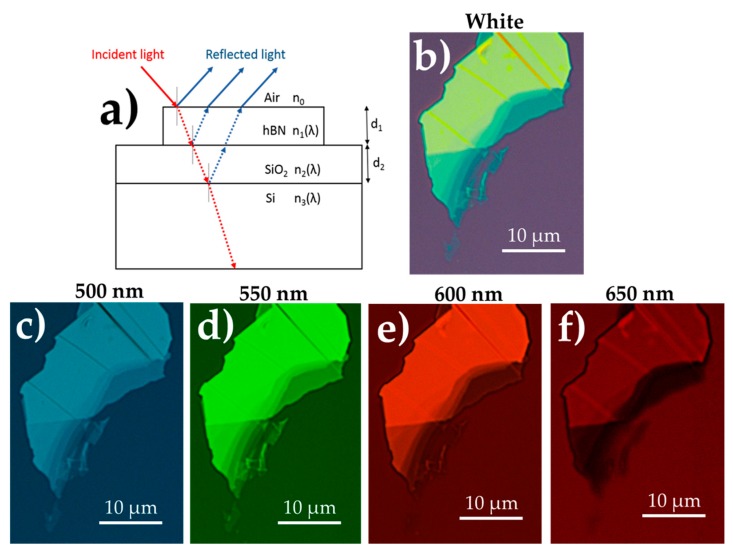
(**a**) Scheme of the hBN/SiO_2_/Si structure with Fresnel multiple reflections for optical contrast identification shown. The Si-substrate is considered semi-infinite with a wavelength dependent refractive index *n*_3_(λ), whereas the SiO_2_ thin film will be characterized by *n*_2_(λ) and thickness *d*_2_. The substrate is covered by hBN microcrystals of refractive index *n*_1_(λ) and thickness *d*_1_, which are surrounded by air with refractive index *n*_0_. Optical images of the same hBN crystal are shown under (**b**) white illumination and (**c**) 500 nm, (**d**) 550 nm, (**e**) 600 nm and (**f**) 650 nm bandpass filter illumination.

Figure 1a shows the scheme of the hBN/SiO_2_/Si multi-layer structure studied in this work. Incident light impinges on this structure through an optical objective, where light suffers from multiple reflections. The reflected light is detected by a charge-coupled device (CCD) sensor. The difference between reflected light from the structure and the SiO_2_/Si substrate results in the visible detection by eye of the hBN micro-crystal. However, we need to maximize the OC signal for a given thickness. For these purposes, the OC can be calculated by using Fresnel equations under normal incident monochromatic light of wavelength λ as a function of hBN and SiO_2_ thicknesses (described in detail in [41]):

(1) $$OC\;\left(\lambda ,d_{1},d_{2\;}\right)=\;\frac{R_{0}\left(\lambda ,d_{1},d_{2\;}\right)-R\left(\lambda ,d_{1},d_{2\;}\right)}{R_{0}\left(\lambda ,d_{1},d_{2\;}\right)}$$

where *R*_0_(λ) and *R*(λ) are the intensities of the reflected light from a SiO_2_/Si substrate and hBN/SiO_2_/Si structure, respectively. In OC calculations, we included the wavelength dependent refractive index and thickness of the different layers of the structure as input (see Figure 1a). Refractive index of the structure varies from 1.85 (400 nm) to 1.78 (700 nm) for hBN (*n_1_*), from 1.48 (400 nm) to 1.46 (700 nm) for SiO_2_ (*n_2_*) and from 5.57 (400 nm) to 3.78 (700 nm) for Si (*n_3_*). The OC contrast was calculated for a SiO_2_ thickness (*d_2_*) of 290 nm, which is in the deviation range of our standard SiO_2_/Si substrate. The thickness (*d_1_*) of test-hBN micro-crystals is defined by the number of monolayers (that multiplied by the thickness of a single monolayer, 0.333 nm [2], gives the value in nm).

Another necessary step in the OC maximization is the modification of OC by the quantum efficiency of the CCD camera sensor. A spectral sensitivity of Bayer filters for red (R), green (G), and blue (B) channels and spectral windows of the bandpass filters are given in Figure S1 of the Supplementary information (SI). By weighting of Equation 1 by the response functions *S*(λ) of the different filters, the theoretical optical contrast, OC_theo_, is [38]:

(2) $$OC_{theo}\;\left(\lambda ,d_{1},d_{2\;}\right)=\frac{\int_{\lambda_{1}}^{\lambda_{2}}S\left(\lambda \right)OC\left(\lambda \right)d\lambda }{\int_{\lambda_{1}}^{\lambda_{2}}S\left(\lambda \right)d\lambda }$$

Equation (2) was then integrated for wavelengths corresponding to electronic B filter (400–500 nm), G filter (500–580 nm), R filter (580–700 nm), and optical bandpass filters at 500 nm (480–520 nm), 550 nm (530–570 nm), 600 nm (580–620 nm), and 650 nm (640–680 nm). The maximal OC_theo_ for a single monolayer (Equation (1)) has a value of 0.018 (1.8%), which is approximately 5 times smaller than for graphene 0.1 (10%) [45] and thus its visual detection is complicated. This is caused mainly by the fact that hBN does not absorb visible light at all. The visibility of a monolayer can be enhanced by using a thinner (≈80 ± 10 nm) SiO_2_ film on top of Si and, simultaneously, by application of a narrow bandpass filter (OC_theo_ = ~3% per monolayer) [43]. The value of OC_theo_ calculated from Equation (2) for a monolayer, for the different filters used here, yields: 1.5% (R), −1.1% (G), −0.6% (B), −1.5% (500 nm), −1.3% (550 nm), 1.4% (600 nm) and 1.3% (650 nm).

Experimentally, we investigated optical images of several hBN microcrystals with different thicknesses by using white illumination (Figure 1b) and the optical bandpass filters (Figure 1c–f). The experimental optical contrast, OC_exp_, was then extracted from these optical images by means of the expression [38]:

(3) $$OC_{exp}\frac{I_{substrate}-I_{sample}}{I_{substrate}}$$

where *I*_sample_ is an average value of pixels occupied by the image of the hBN microcrystal and *I*_substrate_ is an average value of pixels taken in the SiO_2_/Si substrate around the microcrystal.

In order to identify the thickness under white illumination conditions, the optical images were decomposed into the R, G and B channels. Shown in Figure S2 of SI is such decomposition of the microcrystal image shown in Figure 1b, whose thickness varies from 13 monolayers to near bulk-like. The images using the B and G channels provide the best recognition of the hBN microcrystals, whereas that for the R channel does not allow a precise recognition of its stepped thickness profile. Optical images of the same crystal taken with the bandpass filters are depicted in Figure 1c–f in comparison to the white illuminated image (Figure 1b). As can be seen from these images, using the optical bandpass filters of 500, 550 and 600 nm provides a good identification of the hBN profile thickness, which is not the case when using the 650 nm filter, consistent with the aforementioned observations using the electronic RGB filters of the CCD. 

**Figure 2:**
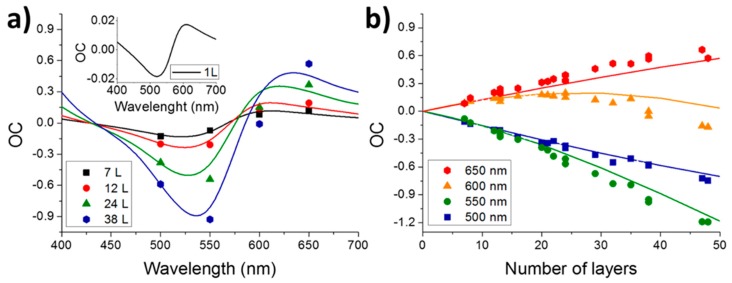
(**a**) Wavelength dependent optical contrast (OC) for hBN thicknesses from 7 to 38 monolayers (L) and a single monolayer (inset) deposited on a 290 nm SiO_2_/Si substrate. (**b**) OC of hBN on a 290 nm SiO_2_/Si substrate as a function of the number of layers. Experimental OC values represent symbols and theoretical lines.

**Figure 3:**
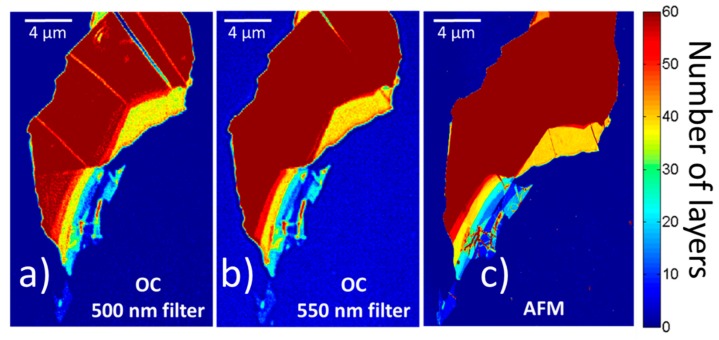
Comparison of optical contrast (OC) images recalculated as function of number of hBN layers obtained by fitting of experimental data for (**a**) 500 nm, (**b**) 550 nm bandpass filter illumination with (**c**) AFM image.

To verify the model, we compared theoretical and experimental data under white illumination and near-monochromatic illumination by using optical bandpass filters. The thickness of test-hBN microcrystals was determined first by AFM. Theoretically and experimentally acquired OC as a function of wavelength for different hBN thicknesses (7–38 monolayers) is plotted in Figure 2a. The experimental and calculated values are in good agreement even for layers as thick as 50 monolayers. In the thickness range explored in this work, the wavelength dependence of OC_theo_ exhibits a negative minimum between 525 and 550 nm (i.e., in this case the hBN microcrystal is brighter than the SiO_2_/Si substrate), whereas OC_theo_ increases by increasing the wavelength and reaches a maximum with positive OC_theo_ (i.e., the hBN microcrystal is darker than the substrate) between 610 and 635 nm. In the OC_theo_ minimum, there is a stronger dependence on the layer thickness in comparison to the case of the OC_theo_ maximum. In the whole wavelength range explored here, OC_theo_ crosses zero twice at a wavelength that is thickness dependent: 427 to 434 nm and 568 to 586 nm. At these wavelengths, few-layer hBN samples on SiO_2_/Si substrates are hardly detectable. We compared the experimental and theoretical values of OC depending on the number of hBN monolayers obtained under white light illumination (Figure S3a in SI) and near-monochromatic illumination using the optical bandpass filters (Figure 2b). The values of OC_exp_ extracted from images, as explained above, increases linearly (positively for the 650 nm filter and negatively for 500 and 550 nm ones, as predicted by OC_theo_(λ) in Figure 1a) up to 50 monolayers of hBN, except in the case of using the 600 nm bandpass filter. This is clearly because this wavelength is very close to the zero-OC value, as shown in Figure 2a. The linear dependence can be used for a rapid identification of the hBN thickness by fitting the experimental data (see Figure S3b,c in SI). Using the corresponding equations acquired by the linear fitting, we recalculated the OC-images registered by using 500 and 550 nm bandpass filters as a function of the number of hBN monolayers and compared them with the AFM image (Figure 3). Clearly, both illuminations are in agreement with the AFM image, proving that the OC-method is appropriate for thickness identification of hBN nanosheets.

### 3.2. Raman Spectroscopy

Raman spectroscopy is an efficient technique for studying crystal quality of 2D materials. The Raman signal is usually thickness dependent and the shift of the Raman peaks (some of them) can be used for thickness identification of atomically thin nanosheets (typically 1–5 layers) [46]. Unlike other 2D materials, such as graphene or some TMDs, the Raman signal of hBN is very weak and only E_2g_ modes are Raman active, which are the analogous to the G-peak in graphene [43]. Two frequency vibration modes can be detected with the E_2g_ symmetry, a low frequency mode with a peak centred at around 52.5 cm^−1^ and a high frequency mode with a peak centered at around 1366 cm^−1^ [37]. The lower frequency mode corresponds to the interlayer vibration and is hardly detectable, which is the reason to use more sensitive Raman techniques, for instance tip enhanced Raman spectroscopy (TERS) or surface enhanced Raman spectroscopy (SERS). The high frequency mode is related to the in-plane vibrations where boron and nitrogen atoms vibrate in opposite directions and its signal is approximately 50 times stronger than the lower frequency one [37,47]. The high-frequency E_2g_ phonon mode could be affected by many factors, such as strain caused by substrate [44], crystal deformations [48,49], defects [50,51], doping [52] or temperature [37,53,54]. In this paper, we concentrated only on the high-frequency E_2g_ phonon mode.

**Figure 4:**
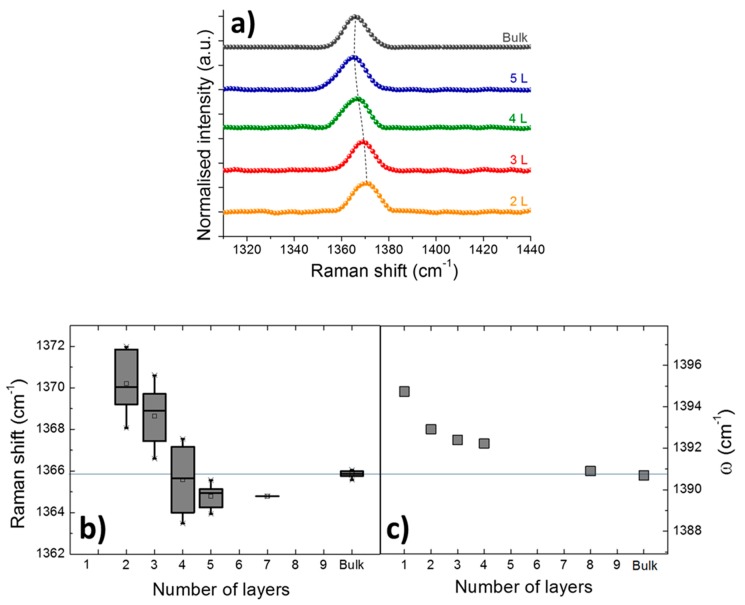
(**a**) Normalized Raman spectra of hBN crystals with thickness varying from 2 to 5 layers and bulk. The spectra are averaged by 6 measurements (2 L), 10 measurements (3 L), 4 measurements (4 L), 14 measurements (5 L) and 19 measurements (bulk). (**b**) Raman shift of the E_2g_ phonon frequencies and (**c**) calculated phonon frequencies (ω) as a function of the number of monolayers. (**b**) is experimentally measured values.

We characterized several hBN microcrystals with thicknesses varying from a single monolayer to near bulk-like. AFM images of atomically thin hBN nanosheets are shown in Figure S4 in SI. Figure 4a shows normalized Raman spectra of hBN crystals representing the average values of measured N nanosheets for 2 L (*N* = 6), 3 L (*N* = 10), 4 L (*N* = 4), 5 L (*N* = 14) and bulk-like (*N* = 19). Figure 4b shows the Raman shift of the E_2g_ phonon mode measured as a function of the nanosheet thickness, as a summary of the Raman spectra registered in more than 40 samples. The E_2g_ phonon model slightly blueshifts by 4 cm^−1^ (with an statistical accuracy not better than 1 cm^−1^) as the thickness of the hBN sample decreases from bulk to the single monolayer, even if the blueshift is observed only below 5 monolayers, as mentioned before. For a better understanding, we performed ab initio calculations to evaluate the layer-dependency of the Raman active phonon modes. The phonon modes are obtained using density-functional perturbation theory (DFPT) within the local-density approximation (LDA), as implemented in QUANTUM ESPRESSO [55]. We used Martins–Troullier norm-conserving pseudopotentials, with an energy cutoff of 90 Ry and k-grid of 12 × 12 × 1, to optimize the lattice parameter. We calculated the Raman active mode for 1, 2, 4, 8 layers and bulk. Figure 4c shows the phonon frequency as a function of the number of layers. Analogously to the experimental frequencies (Figure 4b), the phonon frequency decreases with the number of layers. The difference between the bulk and monolayer frequency obtained in the simulations is 4 cm^−1^, lower than the maximum experimental value of 6 cm^−1^. Note the simulations are performed in ideally suspended layers while boron nitride layers are supported on a SiO_2_/Si substrate. The difference can be attributed to the corrugation induced by the substrate [44].

## 4. Conclusions

In conclusion, we demonstrated that the thickness of hBN microcrysals can be identified through optical contrast measurements. The proposed technique rapidly and effectively distinguishes the thickness of hBN nanosheets with a thickness up to 50 monolayers by using near-monochromatic illumination. We also investigated experimentally and theoretically the evolution of high frequency in-plane E_2g_ phonon peaks as a function of the hBN thickness. Our results show a decrease of the phonon mode frequency with thickness of atomically thin hBN microcrystals. In comparison to values for bulk hBN, we observed an experimental maximum blueshift of 6 cm^−1^ (4 cm^−1^ theoretically) in 2-monolayer thick hBN, with the difference attributed to the corrugation of the nanosheets induced by the substrate. In any case, the small thickness-blueshift of the E_2g_ phonon mode, which is a consequence of its strong in-plane vibration character, makes Raman spectroscopy unsuitable for measuring the thickness of hBN microcrystals thicker than 3 monolayers.

## Supplementary Materials

The following are available online at https://www.mdpi.com/2079-4991/9/7/1047/s1, Figure S1: Relative spectral sensitivity of RGB Bayers filters and spectral windows of the bandpass filters used. Figure S2: White illuminated optical image of a hBN flake and its decomposition to RGB channels. Figure S3: OC of R, G, B channels and band pass filters for SiO_2_ thickness of 290 nm as function of number of hBN layers with shown experimental data extracted from optical images. Figure S4. AFM images of some of measured areas with hBN thickness ranging from 1 to 5 layers.
