# Supplementary material for: Optical Contrast and Raman Spectroscopy Techniques Applied to Few-Layer 2D Hexagonal Boron Nitride
Source: Nanomaterials (Basel). 2019 Jul 22;9(7):1047. doi: 10.3390/nano9071047 (PMC6669639; doi:10.3390/nano9071047)
Supplement: Supplementary file 1 [file nanomaterials-09-01047-s001.pdf]

Supplementary information

Optical contrast and Raman techniques applied to few-layer 2D hexagonal boron nitride

Marie Krečmarová[1], Daniel Andres-Penares[1], Ladislav Fekete[2], Petr Ashcheulov[2], Alejandro Molina-Sánchez[1], Rodolfo Canet-Albiach[1], Ivan Gregora[2], Vincent Mortet[2], Juan P. Martínez-Pastor[1], Juan F. Sánchez-Royo[1]*

[1] ICMUV, Instituto de Ciencia de Materiales, Universidad de Valencia, P.O. Box 22085, 46071 Valencia, Spain; Juan.F.Sanchez@uv.es

[2] Institute of Physics, Academy of Sciences Czech Republic v.v.i, Na Slovance 1999/2, 182 21 Praha 8, Czech Republic; mortetv@fzu.cz

* Correspondence: Juan.F.Sanchez@uv.es; Tel.: +34 96 354 4556

The theoretical optical contrast (OC) was calculated using a response function of Bayers filters of the Nikon DSFI2 camera and bandpass filters. Figure S1 shows a spectral sensitivity of Bayers filters for red (R), green (G), and blue (B) channels and spectral windows of the bandpass filters (500, 550, 600 and 650 nm). On the other hand experimental OC was extracted from captured images and matched with hBN thickness acquired by Atomic force microscopy (AFM). Figure S2 depicts a white illuminated image of a hBN crystal with thickness varying from 13 to bulk-like layers and its decomposition to R, G, B channels. The best recognition of number of layers is achieved by B and G channel OC whereas OC is negligible for R channel. Figure S3a shows comparison of theoretical and experimental OC extracted from R, G, B images for SiO_2 thickness of 290 nm as function of number of hBN layers. The theoretical and experimental data are consistent to each other. Experimental OC values extracted from R, G, B images (Figure S3b), 500 and 550 nm bandpass filters (Figure S3c), 600 and 650 nm bandpass filters (Figure S3c), show quasi-linear dependence up to 50 layers of hBN, except only for illumination by using a 600 nm bandpass filter, the OC shows a polynomial dependency.

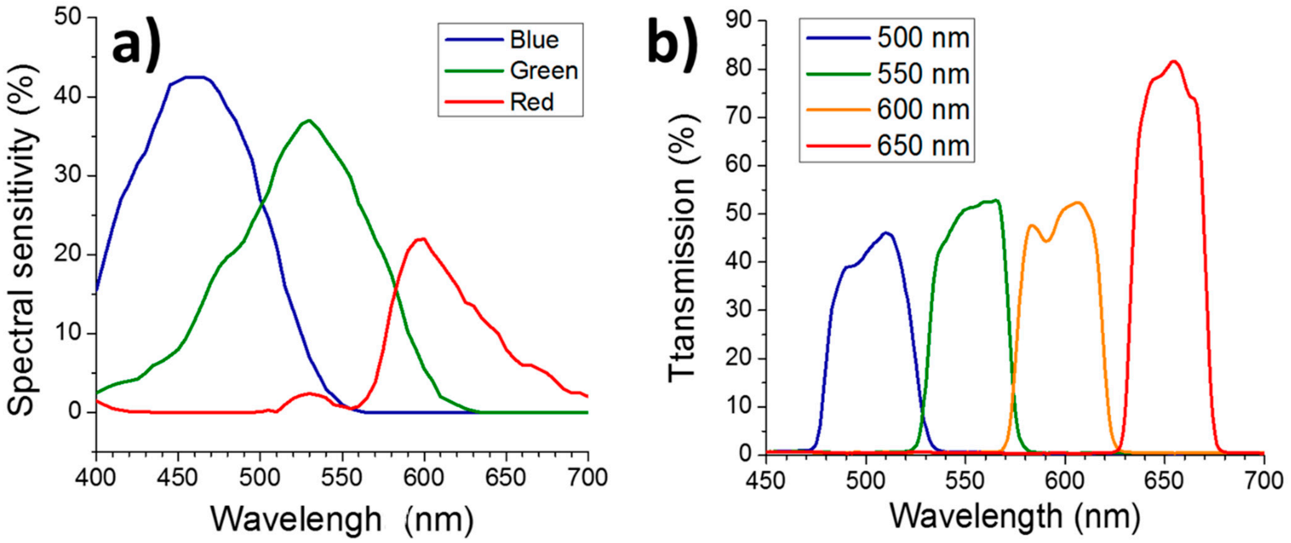

Figure S1. (a) Relative spectral sensitivity of RGB Bayers filters of the Nikon DSFI2 camera. (b) Spectral windows of the bandpass filters. The nominal filter wavelength is indicated.

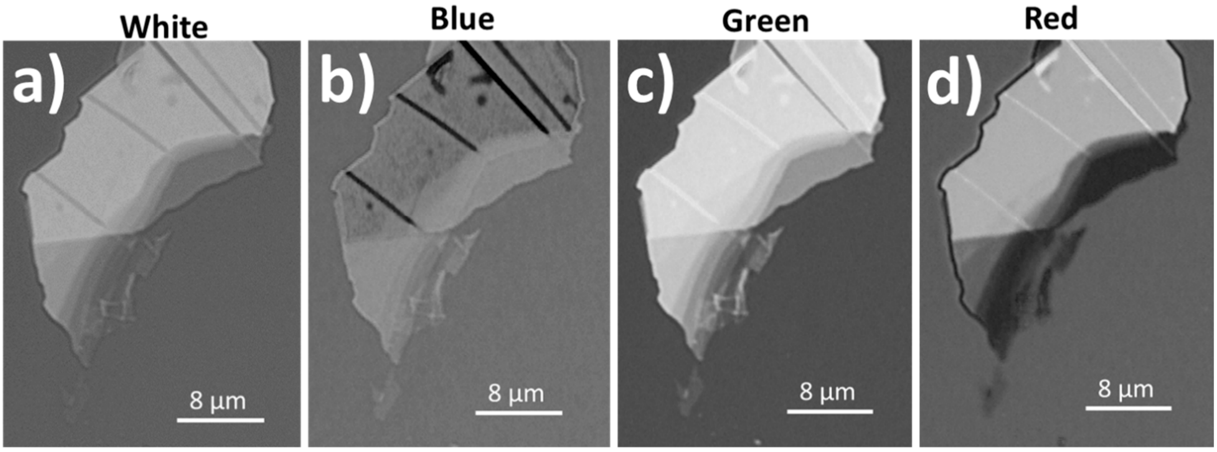

Figure S2. (a) White illuminated optical image of a hBN flake and its decomposition to (b) B, (c) G, (d) R channels.

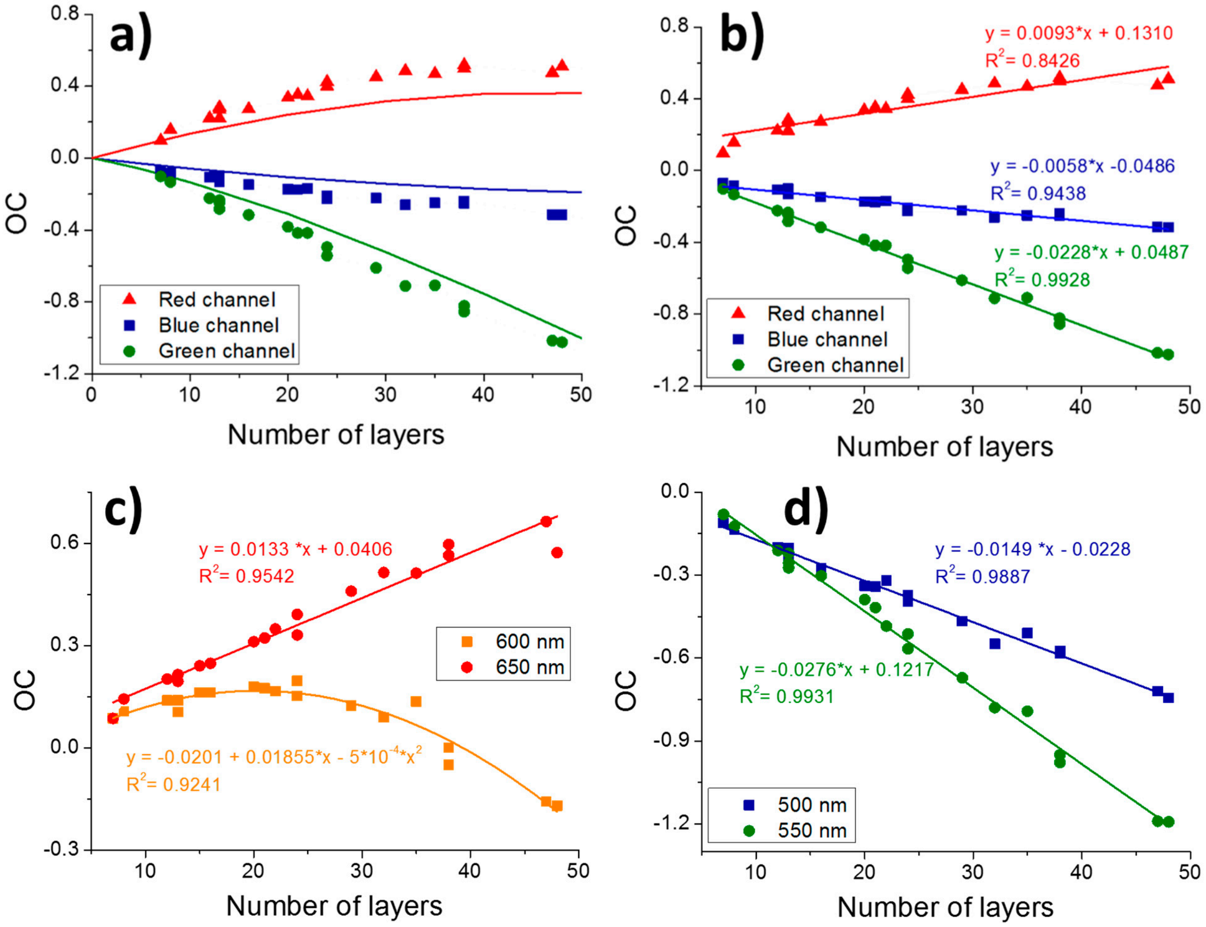

Figure S3. (a) Optical contrast (OC) of red, green and blue channels for SiO_2 thickness of 290 nm as function of the number of hBN layers. Symbols represent experimental and lines theoretical OC values. Experimental data (symbols) extracted from optical images of (b) R, G, B channels, (c) 500, 550 nm and (d) 600, 650 nm bandpass filters. The experimental data were fitted (lines) by linear and polynomial functions with shown equations in insets.

We characterized several hBN crystals with thickness varying from 1 monolayer to bulk-like by atomic force microscopy (AFM), optical contrast (OC), and Raman spectroscopy. Figure S4 shows AFM images of atomically thin hBN crystals with thickness varying from 1 to 5 layers. Some of the exfoliated nanosheets present bubbles and wrinkles.

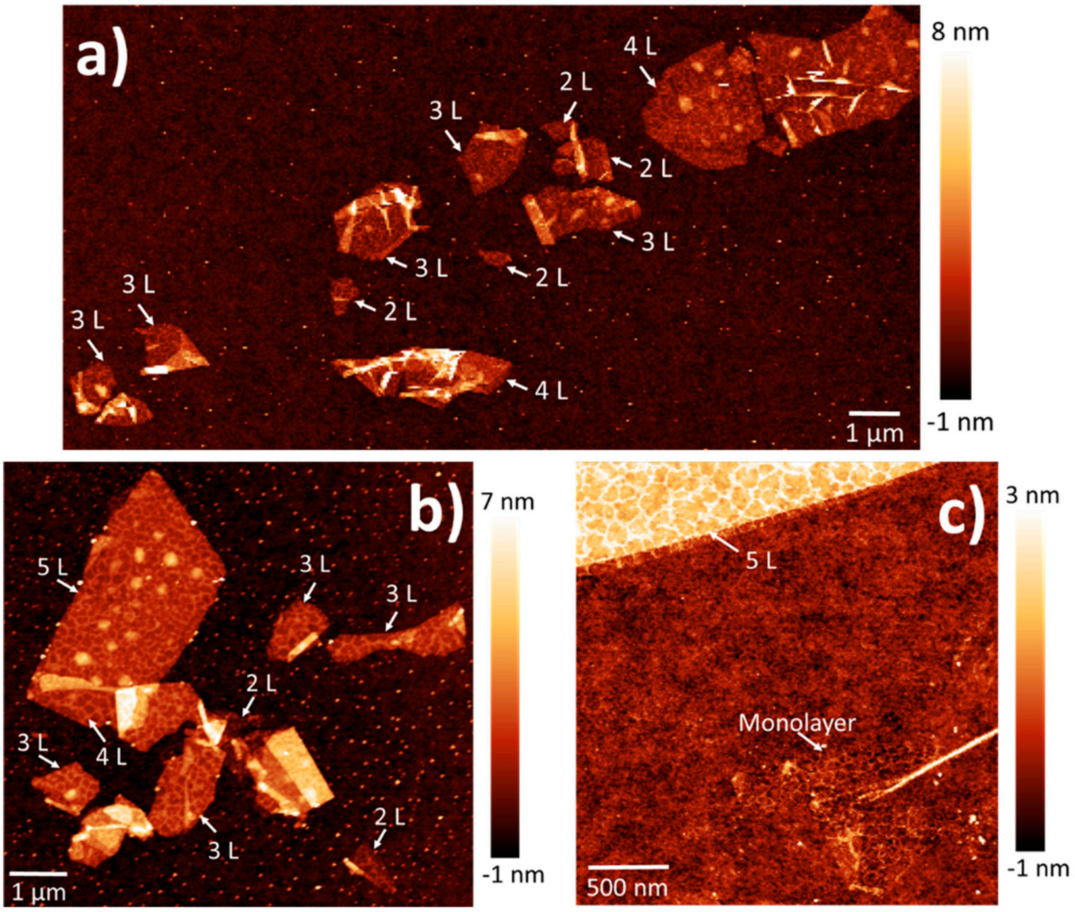

Figure S4. Atomic force microscopy (AFM) images of 3 measured areas a-c with hBN thickness ranging from 1 to 5 layers.
